# Biomarkers in Hereditary Angioedema

**DOI:** 10.1007/s12016-021-08845-6

**Published:** 2021-02-09

**Authors:** Grzegorz Porebski, Mateusz Kwitniewski, Avner Reshef

**Affiliations:** 1 Department of Clinical and Environmental Allergology, Jagiellonian University Medical College, Krakow, Poland; 2 Department of Immunology, Faculty of Biochemistry, Biophysics and Biotechnology, Jagiellonian University, Krakow, Poland; 3 Barzilai University Medical Centre, Ashkelon, Israel

**Keywords:** Hereditary angioedema, Biomarkers, C1 inhibitor, Diagnosis, Management

## Abstract

A biomarker is a defined characteristic measured as an indicator of normal, biologic, pathogenic processes, or biological responses to an exposure or intervention. Diagnostic biomarkers are used to detect a disease or a subtype of a disease; monitoring biomarkers are measured serially to assess a medical condition; response biomarkers are used to check biologic response following a medical intervention; predictive biomarkers are used to identify patients who are more likely to respond to a medical intervention; and prognostic biomarkers are used to assess the future likelihood of a clinical event. Although biomarkers have been extensively investigated and validated in many diseases and pathologies, very few are currently useful for the diagnosis, evaluation of disease activity, and treatment of hereditary angioedema (HAE). Pathophysiologic pathways involved in HAE reveal a plethora of molecules from the complement, coagulation, and fibrinolysis systems or from the vascular endothelium, which may serve as biomarkers. The most promising candidates, together with their laboratory readout systems, should be evaluated with regard to their analytical and clinical validity and utility. To be highly specific, such biomarkers should be linked to the pathomechanisms of HAE, particularly the bradykinin-generating cascade. Additionally, major advances in high-throughput omics-based technologies may facilitate the discovery of new candidate biomarkers in the future. This review will cover the existing as well as future potential biomarkers that will support the diagnosis, monitor disease activity, and can be used to assess the efficacy of new avenues of therapy of HAE and other forms of angioedema.

## Introduction

Biomarkers are currently one of the most extensively investigated areas of biomedical sciences, as reflected by search results in major public databases. A search for the term “biomarker” yields over 1.5 thousand active and recruiting studies in a web-based registry of clinical trials [1]. Moreover, this keyword generates over 40,000 results in the PubMed search (filters applied: clinical trial, humans) [2]. In the field of allergy and clinical immunology, biomarkers have been recently studied in numerous diseases, such as atopic dermatitis [3], allergic rhinitis [4], bronchial asthma [5, 6], food allergy [7], and severe drug hypersensitivity reactions [8]. Most commonly, investigators have searched for new biomarkers that would predict disease severity [3, 5], determine the risk of severe reactions [7, 8], or discriminate between disease endotypes [6].

The main focus of the present review is hereditary angioedema (HAE). The most frequent type of HAE is characterized by recurrent episodes of tissue swelling due to low production or nonfunctional serine protease inhibitor, namely, C1 esterase inhibitor (C1-INH). Affected enzymatic pathways include the classic complement cascade, fibrinolytic pathway, and contact activation system responsible for bradykinin formation. The loss of the inhibitory activity of C1-INH leads to bradykinin overproduction, resulting in vascular instability in the endothelial wall, followed by hyper-permeability and plasma extravasation [9]. Another types of HAE have a similar clinical picture, but with normal C1-INH level and activity and a different genetic background, namely, mutations in the gene-encoding coagulation factor XII (FXII-HAE), plasminogen (PLG-HAE), angiopoietin-1 (ANGPT1-HAE), or an unknown mechanism (U-HAE) [10].

The aim of this article is to review biomarkers that could be used for diagnosis, evaluation of disease activity, and management of these clinical entities.

Scientific literature offers numerous definitions and classifications of biomarkers. To ensure effective communication and consistent use of key terms, we followed the nomenclature proposed by the FDA-NIH Biomarker Working Group [11]. Hence, biomarker was considered as “a defined characteristic that is measured as an indicator of normal biological processes, pathogenic processes, or biological responses to an exposure or intervention, including therapeutic interventions” [11]. For the purpose of this review, we also used the following terms: *diagnostic biomarker* (used to detect a disease or a subtype of a disease), *monitoring biomarker* (measured serially to assess the medical condition of interest), *response biomarker* (used to check if a biological response has occurred in a patient who underwent a medical intervention), *predictive biomarker* (used to identify patients who are more likely to respond to a medical intervention), and *prognostic biomarker* (used to assess the likelihood of a clinical event in the future). Some of these biomarker categories may overlap in particular clinical situations [11]. An ideal biomarker should demonstrate evidence for strong analytical validity and clinical utility, reflect a pathophysiology of a disease, with proven feasibility (an easy and reliable measurement method), and either no or low invasiveness, which implies an easy access to biological material (e.g., blood, saliva, or urine) [12, 13]. The accepted approach to biomarker definition entails the selection of some candidate indicators that are associated with the clinical or pathophysiological aspects of a disease. However, with the advent of novel high-throughput techniques, it is now possible to perform non-targeted multi-omics analyses (e.g., genomic, transcriptomic, proteomic, or metabolomic) to identify a cohort of biomarkers for further in-depth research [12].

In daily practice, clinicians taking care of patients with HAE are faced with several challenges, such as (i) large inter- and intra-familial heterogeneity in the clinical manifestations of the disease; (ii) early diagnosis of unpredictable oncoming angioedema attacks; (iii) a differential diagnosis of HAE subtypes; and (iv) objective assessment of disease severity, which may guide therapeutic decisions. These challenges are the driving force behind the current efforts to identify measurable biomarkers of HAE. Finding such biomarkers could facilitate the diagnosis and prediction of an upcoming attack, as well as help to monitor disease activity, discriminate between high and low responders to a specific drug, or select the best candidates for a given treatment.

This review represents a clinician’s perspective and constitutes an attempt to answer the question: to what extent biomarker discovery can help overcome the above challenges? In the broadest sense, any measurable parameter may serve as a biomarker, including clinical features or radiographic findings, but the scope of this review is to discuss only potential laboratory biomarkers that involve essential biological processes, from nucleic DNA to mediators of effector mechanisms.

## Biochemical Biomarkers

HAE involves numerous processes in pathophysiologic pathways, during remissions and attacks. Recently, plasma enzymatic cascade systems, endothelium-derived factors, and inflammatory mediators have been extensively studied with regards to the mechanistic contributions to the pathology of HAE. Some have important implications for the discovery of biomarkers that could be used in the diagnosis and monitoring [13–17]. However, only a few laboratory tests (antigenic and functional C1-INH, complement C4) with established threshold values are currently used in clinical practice for diagnosis and decision-making [14] (Table 1).

Candidate biomarkers, discussed in detail below, were selected based on the current understanding of the biochemical processes underlying HAE as well as on the evidence for their relevance in clinical disease manifestations.

### Complement Cascade Biomarkers

*Functional C1-INH* (fC1-INH, C1-INH activity) plasma levels are considered a reference test in the diagnosis of HAE due to C1-INH deficiency (C1-INH-HAE), particularly of type 2 [14, 18–20]. However, there have been some concerns in relation to this test, including variability due to sample handling and time of storage or an increase in its levels in the course of inflammation and infection [21–23]. In addition, different types of assays may yield divergent results [19, 24]. Moreover, fC1-INH measurements can be affected by the presence of autoantibodies to C1-INH in the sample [16], as well as by danazol treatment [25] or plasma-derived C1-INH [26]. Currently, fC1-INH levels are measured by a commercial chromogenic assay or an enzyme-linked immunosorbent assay (ELISA), which detects complexes formed by C1-INH and complement C1s, activated FXII, or plasma kallikrein (PKa) [18, 21, 27]. However, functional assays have limited ability in detecting small changes over time [18, 21]. Recently, a highly robust point-of-care test to measure fC1-INH in dried blood spot has been described [28]. It is based on the quantitation of the enzyme reaction product by liquid chromatography–tandem mass spectrometry and is characterized by high reproducibility and accuracy, together with sample storage stability. Whereas the diagnostic value of functional C1-INH levels is well established, its value as a monitoring biomarker remains unclear. A predominant opinion is that the level of fC1-INH has little relationship to the clinical course of C1-INH-HAE [13, 14]. Suffritti et al. showed lower fC1-INH in patients during attacks than during remission (31 and 131 patients, respectively) [29]. However, Kajdasci et al. and Cugno et al. did not confirm this finding in their studies involving 18 and 28 patients with C1-INH-HAE, respectively [30, 31]. Other studies focused on associations between C1-INH function and disease course. Kelemen et al. demonstrated that the baseline level of fC1-INH correlates with the HAE severity score [32]. In a subsequent report by the same group, lower fC1-INH levels were observed in patients with a higher number of attacks and a higher need for C1-INH on-demand treatment [33]. In turn, Bafunno et al. could not find a significant correlation between fC1-INH level and disease severity score or age of onset [34]. Further insight was provided by clinical trials with plasma-derived C1-INH supplementation. A level of approximately 40% of fC1-INH appears to protect against angioedema attacks in most patients who received prophylactic treatment with subcutaneous C1-INH [35], which is in line with previous clinical observations [36, 37]. Therefore, these ranges of fC1-INH may serve as a prognostic biomarker of disease activity, i.e., assessment of likelihood of future attacks.

*Antigenic C1-INH (AgC1-INH)* plasma concentration is a critical diagnostic biomarker for the diagnosis of C1-INH-HAE type 1 [38]. Its concentration can be measured by nephelometry, turbidimetry, or radial immunodiffusion, depending on local availability and cost [23]. The results may be influenced by replacement therapy with C1-INH [14]. In principle, AgC1-INH is not considered a valuable monitoring biomarker of the clinical course of the disease [13, 14, 30, 31]. However, Spath et al. demonstrated most frequent attacks in patients with C1-INH-HAE when AgC1-INH levels were below 0.035 g/l [39]. Other authors reported that AgC1-INH levels were lower during attacks [29] or negatively correlated with the annual number of attacks [33].

*The protease-inhibitor complex C1-INH-C1(r,s)* reflects contact system activation and thus may be considered a potential biomarker [14]. Its plasma concentration, which can be determined by ELISA, is influenced by the amount of C1-INH present in plasma and is artificially low in C1-INH deficiency [14, 33]. Plasma levels of C1-INH-C1(r,s) complexes were found to be higher in patients with C1-INH-HAE than in healthy controls [33, 40] and increased further during angioedema attacks [41]. Patients with higher C1-INH-C1(r,s) levels had a history of more severe attacks and more often required emergency treatment [40]. Plasma C1-INH-C1(r,s) complex levels were reported to normalize in patients treated with stanozolol along with a reduction in symptoms [42], thus showing promise as a biomarker for monitoring therapeutic response (a response biomarker) [43].

*Complement C4* serves as an important contributory diagnostic biomarker in C1-INH-HAE [14, 38], because its level is reduced in most patients, especially during attacks [16, 24]. Nevertheless, its performance as a monitoring biomarker is poor. Complement C4 was shown to correlate with the frequency of attacks and on-demand consumption of C1-INH concentrate [33] but not with disease severity scores [32]. No significant difference was observed in C4 levels between remission and acute abdominal attacks [31].

Additional laboratory complement indices were also reported in HAE. Varga et al. found that *anti-C1-INH IgM* antibody levels correlated with disease severity in C1-INH concentrate–naive patients [44]. Other reports indicated that the levels of *Mannose-binding lectin-associated serine protease*s *(MASP-1, MASP-1)-C1-INH complexes* are lower in C1-INH-HAE patients and correlate with the frequency of attacks [45], whereas the levels of *MASP-2 and ficolin-3/MASP-2 complexes* increase during attacks [46] (Table 1).

### Contact System and Bradykinin-Forming Cascade Biomarkers

Since *bradykinin (BK)* is the major mediator of swellings in HAE [13, 47], it is expected to be the most accurate biomarker of upcoming attacks. Indeed, plasma BK levels were reported to be higher in patients with C1-INH-HAE than in healthy controls, with a further significant rise during attacks [48]. Moreover, they were found higher in blood taken from the site of angioedema than at a control site in classical report by Nussberger et al. [49]. Nevertheless, clinical utility of plasma BK levels is being questioned because of its high sensitivity to pre-analytical procedures and a very short half-life (measured in seconds) [13, 14]. In addition, the measurement of BK and its breakdown metabolites, such as *des-Arg-BK*, based on liquid chromatography with mass spectrometry, is technically challenging [50, 51]. The clinical utility of a commercially available assay kit for detecting the product of BK degradation, which might be a surrogate for BK quantification, awaits confirmation [13].

*High molecular weight kininogen (HK)* proteolysis by active PKa results in generation of *cleaved HK (cHK)* and BK. Thus, cHK is thought to be a promising indirect marker of BK release and contact system activation, which occurs during HAE attacks. Western blotting for cHK detection is hard to standardize and subjective in interpretation. Semi-quantification of results can be done by scanning gels [13]. New effective methods for cHK measurement are based on a monoclonal antibody ELISA [52] and modification of liquid chromatography–mass spectrometry [53]. Cugno et al. observed, in patients with C1-INH-HAE, high levels of cHK during attacks and normal levels during remission [54, 55]. In a subsequent report on a large population of C1-INH-HAE patients, Suffritti et al. reported cHK levels to be higher in patients than in controls and to further increase during attacks. They also demonstrated that cHK levels were higher in highly symptomatic patients in comparison with those with less frequent attacks [29]. Using an immunoassay based on ELISA, Hofman et al. confirmed that cHK levels were elevated in patients with C1-INH-HAE during remission and further increased during attack [52]. Banerji et al. showed significant reductions in cHK levels in C1-INH-HAE patients treated with 300 mg and 400 mg of lanadelumab, a monoclonal antibody against PKa [56]. In turn, Bova et al. measured cHK levels in a cohort of 105 HAE patients with normal C1-INH levels (nC1-INH-HAE), including U-HAE and FXII-HAE [57]. cHK was measured during remission in plasma collected with and without using protease inhibitors. In patients with U-HAE, cHK levels were similar to those in healthy controls with the use of protease inhibitors and significantly increased without them. In patients with FXII-HAE without the use of protease inhibitors, cHK levels were higher than in controls and similar to those observed in patients with U-HAE [57].

Therefore, cHK levels can discriminate between patients and healthy individuals, between highly symptomatic and less symptomatic patients, and between acute attack and remission. The development of a new reliable and less laborious measurement method may facilitate the clinical application of this parameter.

*Plasma kallikrein (PKa)* is also considered a potential biomarker of BK-mediated angioedema attacks. Spontaneous PKa activity, measured with the use of a chromogenic substrate, was higher in patients with C1-INH-HAE than in controls and further increased during attacks [29]. Similar results were reported by other authors [58]. Lara-Marquez et al. developed an assay based on plasma capacity to generate active PKa under ex vivo stimulation with dextran sulfate [59]. Using different threshold cutoff values in this assay, patients with BK-dependent angioedema (nC1-INH-HAE and C1-INH-HAE) could be distinguished from controls without swelling attacks and patients with histaminergic angioedema, based on a higher amount of generated PKa [59].

*Activated coagulation factor FXII (FXIIa)* was shown to be higher in patients with C1-INH-HAE than in healthy controls, with a further increase during attacks [54, 58, 60]. The activity of FXII was also evaluated as a possible biomarker of FXII-HAE in symptom-free periods, but studies provided inconsistent results, with some suggesting an increase while others reporting no difference between patients and healthy controls [61, 62].

*Factor XIIa/C1-INH complexes* were studied by Konings et al. [63]. They showed that the levels of these complexes, as well as the levels of FXIa-C1INH and PKa-C1INH complexes, were lower in patients with C1-INH-HAE than in healthy controls after an in vitro activation of the samples with an FXII trigger.

*Kinins*—degradation and accumulation of *kinins*, including BK, may modify the clinical phenotype of HAE. *Carboxypeptidase N (CPN)*,* angiotensin-converting enzyme (ACE)*, and *aminopeptidase P (APP)* are major kinases involved in kinin catabolism [64]. Drouet et al. investigated the *kininase* activity in C1-INH-HAE patients with and without androgen prophylaxis [65]. APP levels were inversely correlated with disease severity in all patients, and CPN activity showed the same relationship only in untreated patients. Subsequently, a similar study was conducted in patients with FXII-HAE, showing an inverse correlation between the disease severity score and both ACE and CPN activities, but not APP [66]. Moreover, the total activity of serine proteases was shown to be higher in patients with C1-INH-HAE and nC1-INH-HAE than in healthy controls and to further increase in those with nC1-INH-HAE during attacks [67].

### Coagulation and Fibrinolytic Pathway Biomarkers

Coagulation and fibrinolysis are also pathways activated in HAE and therefore extensively investigated for potential biomarkers. To date, a wide range of *fibrinogen split products* in patients during HAE attack and remission have been studied [31, 55, 60, 68, 69]. The findings from these studies are summarized in Table 1.

*Plasminogen activator inhibitor (PAI)-1* levels, *prothrombin time*, and *activated partial thromboplastin time *were also found to be lower during C1-INH-HAE attacks compared with a symptom-free period [60]. The PAI-1 level was also found lower in patients with U-HAE and FXII-HAE compared with control individuals, but the difference was not significant [70]. In the same study, also *PAI-2 levels* were reported to be lower in patients with U-HAE and FXII-HAE than in controls, but this finding was not corroborated by other studies [71].

*D-dimer* levels were also found elevated and may also discriminate between abdominal HAE attacks and abdominal colic episodes [31, 72], between multiple- and single-site attacks [60], and between submucosal (abdominal, oropharyngeal–laryngeal) and subcutaneous (peripheral, facial) attacks [73]. D-dimer levels were observed to decrease at day 7 after attack [72, 73]. Prophylaxis with continuous supplementation of subcutaneous plasma-derived C1-INH seems to suppress D-dimer levels, in parallel with a significant reduction in the rate of HAE attacks [74].

Collectively, laboratory parameters associated with coagulation and fibrinolysis may have limited usefulness as biomarkers because of their interpatient variation in the plasma [13]. They can be useful to some extent for disease course monitoring when compared with baseline values of the same individual. Another major disadvantage that limits their potential use as biomarkers is the fact that they may not be specific for HAE. For instance, a D-dimer test is widely used and easily available in emergency settings, but D-dimer levels are elevated in numerous medical conditions, i.e., pulmonary embolism and venous thromboembolism, but also in infection, autoimmune disorders, malignancy, as well as in smokers, pregnant women, and elderly individuals [75].

### Endothelium-Associated Biomarkers

As the endothelium is inherently related to microvascular permeability and, in consequence, the swelling phenomenon, it represents another area of interest regarding potential biomarkers in HAE. Studies in this field involved vascular endothelial *cadherin* (transmembrane adhesive protein) [72, 76], *von Willebrand factor* (marker of endothelial damage), *soluble E-selectin* (cytokine-induced adhesion molecule), *endothelin-1* (vasomotor activity regulator) [30, 77, 78], *arginine vasopressin*, *adrenomedullin* [78], *atrial natriuretic peptide* [79], as well as *endothelial-derived endocan* and vascular cell *adhesion molecule-1* (markers of endothelial function) [80]. The subsequently studied modulators of vascular permeability included *vascular endothelial growth factors*,* angiopoietin-1* (which promotes endothelial stabilization) and *angiopoietin-2* (which facilitates vascular permeability) [81, 82], secreted *phospholipases A2* (particularly the 2A group) [83], and *platelet-activating factor acetylhydrolase* [81]. Studies investigated changes in the above factors in small groups of patients with C1-INH-HAE during attacks and symptom-free periods, in comparison with healthy controls, as shown in Table 1. More recent research also involved patients with nC1-INH-HAE in remission. Bova et al. reported increased levels of *angiopoietin-1 and vascular endothelial growth factors A and C* in patients with U-HAE as well as increased levels of vascular *endothelial growth factor C* in patients with FXII-HAE [57].

It is well known that stimulation of endothelial cells with BK and its analogs causes the release of *prostacyclin* and unstable *endothelium-derived relaxing factor* [84], which was subsequently identified as *nitric oxide species *contributing to enhanced vascular permeability [85]. Bas et al. investigated the long-lived metabolite of *prostacyclin*, namely, 6-keto-prostaglandin F1-α, as a potential biomarker for the diagnosis of angioedema induced by angiotensin-converting enzyme inhibitors [86]. In turn, Demirturk et al. showed that plasma levels of endothelial *nitric oxide synthase* were significantly higher in patients with C1-INH-HAE in remission and during attacks than in healthy participants and that the levels of nitric oxide metabolites were elevated only during attacks [87]. Further research involved also the effect of HAE on endothelial function expressed by blood concentrations of *asymmetric* *dimethylarginine*, a strong inhibitor of nitric oxide synthesis associated with numerous common conditions, such as atherosclerosis. In a group of HAE patients (24 with C1-INH-HAE and 14 with FXII-HAE), *asymmetric dimethylarginine* levels were higher than in controls [88]. In a subsequent report, this group demonstrated that serum concentrations of advanced oxidation protein products, used as markers of oxidative stress, were higher in patients with C1-INH-HAE and FXII-HAE during remission than in controls [89]. In another study investigating oxidative stress in HAE, the authors showed that the levels of *reactive oxygen species* in peripheral blood mononuclear cells of patients with C1-INH-HAE were higher than in controls; however, no differences were observed between the groups in plasma levels of advanced oxidation protein products [90].

### Other Areas of Research

A number of studies have investigated the association of HAE with low-grade inflammation markers, immune system elements, and hormones. Investigators compared patients with C1-INH-HAE and healthy controls with respect to a number of factors, including *C-reactive protein*, *erythrocyte sedimentation rate*,* white blood cell*, and *neutrophil counts* [91–93], a wide network of pro-inflammatory and anti-inflammatory cytokines (i.e., *interleukins IL-1β*,* IL-2*,* IL-4*,* IL-5*,* IL-6*,* IL-8*,* IL-10*,* IL-13*,* IL-17*,* interferon-γ*,* tumor necrosis factor-α*,* granulocyte colony-stimulating factor*, and* granulocyte-macrophage colony-stimulating factor*) [87, 94, 95], as well as *sex hormones*,* such as progesterone*,* together with sex hormone-binding globulin* [96]. Differences in these factors between patients during remission and attacks, as well as in comparison with healthy controls, are summarized in Table 1. More recently, significant changes were shown in multifunctional *human glycoprotein fetuin-A* levels between symptom-free periods and attacks in patients with C1-INH-HAE [93]. Moreover, fragmentation patterns of serum *glycoprotein 120* were also reported as a potentially useful biomarker in patients with nC1-INH-HAE [97] (Table 1).

The utility of biomarkers reported in the above studies should be interpreted with caution if (i) biochemical parameters investigated in the study are involved also in other clinical pathologies (low specificity in HAE, uncertain direction of a causative relationship between the studied laboratory parameter, and HAE); (ii) the study includes a small sample size of patients; and (iii) results of laboratory measurements are highly variable. For biomarkers expected to facilitate the monitoring of HAE severity or response to treatment, well-defined endpoints are necessary. However, differences in symptom severity scores used and lack of standardization make it difficult to compare the results between studies. In turn, potential diagnostic biomarkers for HAE or angioedema attacks require a gold standard for their validation. Hence, a new test based on the measurement of a given molecule has to be validated against proven clinical events or cases (e.g., an investigator-confirmed attack or a known genetic mutation with proven pathogenicity). Studies designed to elucidate the mechanisms of HAE usually are unable to strictly follow the above requirements for biomarker identification, but there are already some ongoing trials directly dedicated to the study of biomarkers in HAE, including specific biomarkers of BK-mediated angioedema attack in a pediatric population, potential disease-specific biomarkers for HAE with the use of dry blood spot technology, and, finally, the use of cHK as a biomarker in C1-INH-HAE [1].

## Genomic Biomarkers

Genomic biomarkers include DNA sequence variations, such as single-nucleotide variants, insertions, and deletions, as well as RNA alterations, such as differential gene expression and micro RNAs [98]. Genetic testing of angioedema is primarily focused on detecting alterations in the DNA of genes encoding proteins that are part of the complement, fibrinolysis, coagulation, kinin, and vasculature systems, including C1-INH (*SERPING1*), FXII (*F12*), plasminogen (*PLG*), or angiopoietin-1 (*ANGPT1*). Genomic biomarkers are mainly employed to support the diagnostic workup for HAE, but efforts are being made to identify correlations of the detected genetic changes with disease severity and treatment outcomes (prognostic or predictive biomarkers) [99].

## Diagnostic Applications of Genetic Biomarkers

The most common form of hereditary angioedema, C1‐INH‐HAE, is primarily caused by alterations in the *SERPING1* gene. Nevertheless, *SERPING1* genotyping is not recommended for the diagnosis of C1-INH-HAE, with some exceptions (e.g., newborns or inconclusive biochemical tests), because biochemical C1-INH testing is cost-effective and reliable [100, 101].

Genotyping is required for the diagnosis of nC1-INH-HAE [100, 102]. It is a rare type of HAE associated with mutations in *F12* [103], *PLG* [104], and *ANGPT1* [105] genes. However, it is estimated that the genetic background of approximately 70% of patients with nC1-INH-HAE is unknown [106]. The *F12* gene, encoding human FXII, is located on chromosome 5 and consists of 13 introns and 14 exons [107]. To date, four *F12* alterations have been linked to nC1-INH-HAE, and all are located on exon 9. Two missense mutations result in the substitution of threonine to arginine or lysine at position 309 of FXII (p.Thr309Lys/Arg) [103]. These two mutations are responsible for approximately 15% to 30% of nC1-INH-HAE cases [100]. In addition, a deletion of 72 bp (c.971_1018 + 24del72) [108] and a duplication of 18 bp (c.892_909dup) [109], affecting the same region of the FXII protein, were identified. Therefore, only exon 9 of *F12* should be investigated as a routine molecular diagnostic biomarker of FXII-HAE [100].

More recently, new mutations in *ANGPT1* [105, 110], *PLG* [104, 111], *KNG1* (Kininogen 1) [112], and *MYOF* (myoferlin) [113] genes were reported in nC1-INH-HAE. However, the importance of these genetic changes in the pathophysiology of HAE is not yet well understood. Several loss-of-function mutations have been identified in the *ANPGPT1* gene-encoding angiopoietin-1 (ANGPT1-HAE). The p.Ala119Ser substitution affects binding of the protein to the tyrosine kinase Tie2 receptor on endothelial cells [105], and, in turn, may reduce the ability to counteract the changes in vascular permeability induced by a variety of mediators, including BK or vascular endothelial growth factor [110]. Other potentially pathogenic variants of *ANGPT1* (p.Ala8Val; p.Gln370His) were found by Cagini et al. [114]; however, more evidence supporting these preliminary results is needed. To date, one missense mutation in the *PLG* gene, which encodes plasminogen, has been linked with nC1-INH-HAE (PLG-HAE). The substitution of lysine to glutamic acid at position 311 of mature plasminogen protein was described independently by Bork et al. [104] and Dewald et al. [111]. It was suggested that altered structure of plasminogen may affect the affinity to its binding partners [111]. The links between plasminogen activation and angioedema were revised in detail by Maas [115]. The coincidence of multiple mutations in the genes of the complement, fibrinolysis, coagulation, and kinin systems, as well as their role in HAE development, is still the subject of debate. Patients harboring both *SERPING1* and *PLG* mutations were described by Bork et al. [116]. Therefore, there is a strong need for a polygenic diagnosis of nC1-INH-HAE.

For some time now, whole-exome sequencing (WES) has been widely used to uncover a molecular background and complex genetic interactions in nC1-INH-HAE. Bork et al. [112] applied it to identify a novel variant of the *KNG1* gene, resulting in the substitution of methionine to lysine at position 379 of the high-molecular-weight kininogen protein (p.Met379Lys). The amino acid change may affect the formation of BK from this protein and was considered to be likely pathogenic. WES has been recently used in multiple studies, and myoferlin (*MYOF*; p.Arg217Ser) [113] has been linked with nC1-INH-HAE. However, further studies are needed to elucidate the role of these genes in HAE and their potential use as diagnostic biomarkers. To date, no specific guidelines for the diagnosis of the above types of angioedema have been developed. The exclusion of the above pathogenic mutations in patients with normal fC1-INH levels makes it possible to establish the diagnosis of U-HAE and non-histaminergic angioedema [100].

## Prognostic Applications of Genetic Biomarkers

The severity and course of HAE may vary greatly even among family members harboring the same mutation [117]. This could be explained, at least in part, by the type of alterations in the *SERPING1* gene and mutations in other genes encoding proteins that are part of the complement, fibrinolysis, coagulation, and kinin systems [99]. The involvement of epigenetic changes [118], viruses, and colonizing microorganisms [119], as well as environmental factors in the pathogenesis of HAE, has also been postulated [61]. As genotype-phenotype correlations in HAE have been reviewed in detail by Loli-Ausejo et al. in the current issue, we only provide a few representative examples of studies with important implications for the discovery of prognostic biomarkers. Several studies investigated correlations between the type of mutations in the *SERPING1* gene (including nonsense, frameshift, large deletions or insertions, splicing defect, and missense mutations) and the clinical course of C1‐INH‐HAE. Only patients carrying missense mutations leading to the change of a single amino acid exhibited a less severe clinical phenotype [120–122]. However, the results concerning the effect of these mutations on the onset of HAE were conflicting. Bors et al. [120] and Speletas et al. [121] found that the first symptoms of C1‐INH‐HAE appeared at an older age in patients harboring missense mutations. Several other studies did not find a correlation between different types of mutations and clinical phenotype, but the size of the patient population was relatively small [34, 123, 124].

Disease-modifying factors, such as *F12* or *KLKB1* gene polymorphisms, have been studied in the context of the clinical variability of C1‐INH‐HAE or nC1-INH-HAE. The c.-4C/T polymorphism (rs1801020) in the 5-UTR region of the *F12* gene was associated with a significantly delayed disease onset [120, 125, 126], regardless of the type of *SERPING1* mutations [125]. Moreover, the T allele was more common in asymptomatic patients [127]. An association between the c.428G/A (rs3733402) polymorphism in the *KLKB1* gene, encoding PKa, and clinical variability of C1‐INH‐HAE was also investigated. Gianni et al. [128] showed that patients carrying G allele exhibited a delayed onset of HAE. The disease onset was further delayed in individuals harboring both c.-4C/T and c.428G/A polymorphisms. No associations were found between the type of *SERPING1* mutations and age at disease onset. More recently, the F12 c.-4C/T polymorphism has been linked with FXII-HAE (p.Thr309Lys variant). Patient carrying the c.-4CC genotype showed higher PKa-like activity and exhibited more severe and frequent manifestations of the disease [129].

## Future Research Directions

**Fig. 1:**
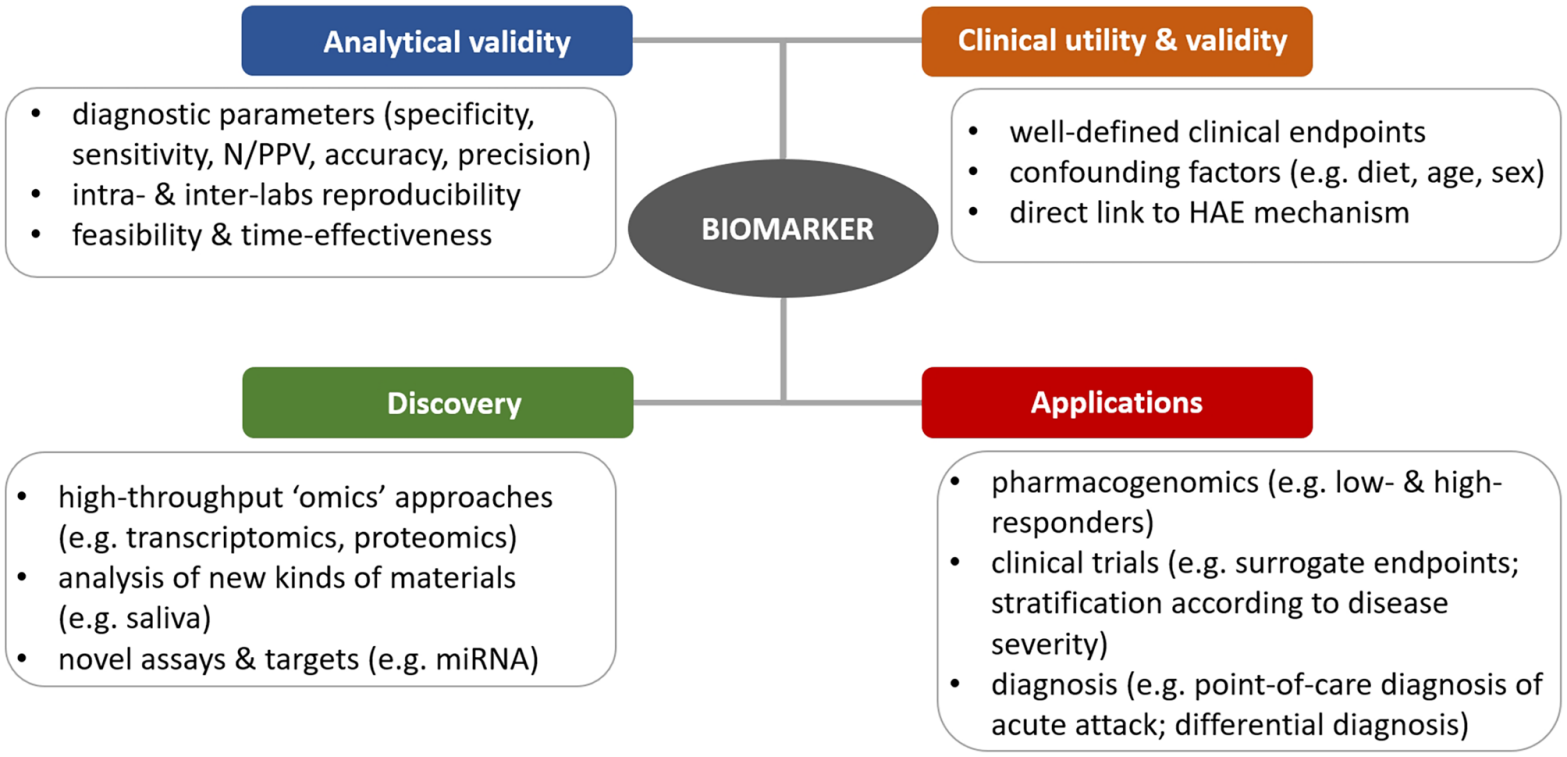
Biomarkers in hereditary angioedema—future applications and research directions. NPV/PPV, negative/positive predictive value; HAE, hereditary angioedema; miRNA, micro RNA

Future applications and research directions in the field are summarized in Fig. 1. A combination of several different biomarkers can also be used. Such an approach may increase overall performance, especially when investigated biomarkers reflect different underlying pathways of HAE. Pharmacogenomic predictive biomarkers might identify individuals who are or are not likely to respond to treatment and thus guide the use of a targeted therapy. Therefore, a discovery of such potential biomarkers would have important implications for therapeutic recommendations reflecting the precision medicine paradigm (“the right drug to the right patient”) [12]. Moreover, biomarkers may serve as validated surrogate endpoints in clinical trials if there is a strong mechanistic rationale and clinical evidence for a possible correlation between a change in biomarker levels and a specific clinical endpoint [11]. Furthermore, validated biomarkers could provide consistent and objective outcome measure and thus facilitate a comparison of different intervention strategies and clinical trial results.

## Conclusions

In conclusion, validated biomarkers could enable a precise diagnosis and personalized management as well as facilitate clinical trials in HAE. New technologies may help establish such biomarkers together with laboratory tests for their detection and measurement, but there is an urgent need to conduct more well-designed studies or complete the ongoing ones in order to obtain solid evidence for their usefulness.

**Table 1:** Laboratory measures discussed in the review, which are considered as potential biochemical HAE biomarkers in literature

System involved^a^	Correlation with disease severity^b^	Increase or further increase during attacks (vs during remission)	Higher level in remission (vs healthy controls)	Lower level in remission (vs healthy controls) or decrease during attacks (↓)	References
Complement	*C1-INH function* C1/C1-INH complex Anti-C1-INH IgM antibodies MASP-1 MASP-1/C1-INH complex	C1/C1-INH complex MASP-2 MASP-2/ficolin-3 complex	C1/C1-INH complex	*C1-INH function * *C1-INH concentration* *C4* MASP-1 MASP-1/C1-INH complex	[14, 16, 18-21,33-38, 40-42, 44-46]
Contact activation and bradykinin	Cleaved HK APP ACE and CPN (3)	Bradykinin Cleaved HK Plasma kallikrein Factor XIIa	Bradykinin Cleaved HK (1)(2) Plasma kallikrein Factor XIIa		[30, 48, 52, 54, 55, 57, 58, 60, 65]
Coagulation and Fibrinolysis		*D-dimer* Prothrombin fragments 1 + 2 TAT, PAP complexes Thrombin, TAFI PAI-1	*D-dimer* Prothrombin fragments 1 + 2 TAT, PAP complexes Factor XIa	PAI-1 ↓ *Prothrombin time ↓* *aPTT ↓*	[32, 55, 60, 68, 69, 72-74]
Endothelium factors	VEGF-A^c^, VEGF-C^c^ ANGPT2^c^	VE-cadherin (soluble form) VWF antigen VWF collagen-binding activity Soluble E-selectin Endothelin-1 Arginine vasopressin Adrenomedullin ANGPT1 eNOS, NO metabolites ADMA (4)	Soluble E-selectin Endocan (soluble form) VCAM-1 (soluble form) sPLA_2_ activity VEGF-A (1), VEGF-C (1)(2) ANGPT1 (1) ANGPT2 PAF-AH eNOS AOPPs (2) ROS	Atrial natriuretic peptide ANGPT2/ANGPT1 ratio ↓ sPLA_2_ activity ↓ PAF-AH ↓	[31, 72, 77-83, 87-90]
Other	Progesterone, SHBG	CRP, ESR, WBC Neutrophil count, neutrophil elastase, myeloperoxidase, pentraxin 3 Fetuin-A TNF-α IL-1β, IL-4, IL-5, IL-6, IL-8, IL-13, IL-17 FGFb, G-CSF, GM-CSF	WBC Neutrophil count GM-CSF, FGFb, IL-17 sgp120 fragmentation (5)	Fetuin-A TNF-α	[87, 91-97]

Data in the table concern HAE, unless otherwise indicated: (1) also in U-HAE, (2) also in FXII-HAE, (3) in FXII-HAE, (4) in group consisting from C1-INH-HAE and FXII-HAE patients, (5) also in nC1-INH-HAE incubated at 4 °C in plastic. Italics: currently in use diagnostic biomarkers; ↓ decrease during attacks

*ACE* angiotensin-converting enzyme, *ADMA* asymmetric dimethylarginine, *ANGPTs* angiopoietins, *AOPPs* advanced oxidation protein products, *APP* aminopeptidase P, *aPTT* activated partial thromboplastin time, *C1-INH* C1-inhibitor, *HK* high molecular weight kininogen, *CPN* carboxypeptidase N, *CRP* C-reactive protein, *eNOS* endothelial nitric oxide synthetase, *E-selectin* endothelial selectin, *ESR* erythrocyte sedimentation rate, *FGFb* basic fibroblast growth factor, *G-CSF* granulocyte colony stimulating factor, *GM-CSF* granulocyte-macrophage colony stimulating factor, *ILs* interleukins, *MASP* mannose-binding lectin-associated serine protease, *NO* nitric oxide, PAF-AH platelet-activating factor acetylhydrolase, PAI-1 plasminogen activator inhibitor-1, PAP plasmin-anti-plasmin complexes, ROS reactive oxygen species, sgp120 serum glycoprotein 120, SHBG sex hormone-binding globulin, sPLA2 secreted phospholipase A2, TAFI thrombin-activatable fibrinolysis inhibitor, TAT thrombin/antithrombin complex, TNF tumor necrosis factor, VCAM vascular cell adhesion molecule, VE-cadherin vascular endothelial cadherin, VEGF vascular endothelial growth factor, VWF von Willebrand factor, WBC white blood cell count

^a^Pathophysiologic pathways of the systems may partially overlap

^b^Disease severity is defined in different ways in particular publications

^c^Higher plasma levels in patients with > 12 attacks/year than in other patients
